# Supplementary material for: Neuroendocrinology of the lung revealed by single-cell RNA sequencing
Source: eLife. 2022 Dec 5;11:e78216. doi: 10.7554/eLife.78216 (PMC9721618; doi:10.7554/eLife.78216)
Supplement: Supplementary file 1. [file elife-78216-supp1.docx]

**Table S1. Mouse airway neuroendocrine cell markers**

| \|  \| **Fraction of expressing cells** \| \| **Relative**  **expression level** \| **Expression difference**  **(NE/non NE)** \| **TNEC marker (Montoro et al)^5^** \| **TNEC marker (Plasschaert et al)^6^** \| **PNEC-selective marker^7^** \| \| --- \| --- \| --- \| --- \| --- \| --- \| --- \| --- \| \| **Gene^1^** \| **NE** \| **non NE^2^** \| **ln (NE/non NE)^3^** \| **p-value^4^** \|  \|  \|  \| \| Pcsk1^1^ \| 0.96 \| 0.01 \| 7.5 \| ≤ 0.0001 \| + \| + \|  \| \| Resp18^1^ \| 0.99 \| 0.03 \| 7.7 \| ≤ 0.0001 \|  \|  \|  \| \| Nov \| 0.99 \| 0.04 \| 7.2 \| ≤ 0.0001 \| + \| + \|  \| \| Piezo2 (Fam38b)^1^ \| 0.96 \| 0.02 \| 6.8 \| ≤ 0.0001 \|  \|  \| + \| \| Scg5^1^ \| 0.96 \| 0.02 \| 6.5 \| ≤ 0.0001 \| + \| + \|  \| \| *Calca^1^ \| 0.93 \| 0.02 \| 7.0 \| ≤ 0.0001 \| + \| + \|  \| \| Ptprn^1^ \| 0.90 \| 0.02 \| 5.9 \| ≤ 0.0001 \|  \|  \| + \| \| Col8a1^1^ \| 0.82 \| 0.01 \| 6.4 \| ≤ 0.0001 \|  \|  \|  \| \| Nnat^1^ \| 0.98 \| 0.11 \| 5.4 \| ≤ 0.0001 \|  \|  \|  \| \| Snap25^1^ \| 0.78 \| 0.01 \| 6.1 \| ≤ 0.0001 \| + \| + \|  \| \| Pkib^1^ \| 0.83 \| 0.03 \| 2.8 \| ≤ 0.0001 \| + \| + \|  \| \| Spock3^1^ \| 0.77 \| 0.00 \| 6.3 \| ≤ 0.0001 \|  \| + \|  \| \| Slc35d3^1^ \| 0.90 \| 0.05 \| 4.3 \| ≤ 0.0001 \|  \| + \|  \| \| Ascl1^1^ \| 0.74 \| 0.00 \| 6.0 \| ≤ 0.0001 \| + \| + \|  \| \| Cplx2^1^ \| 0.91 \| 0.07 \| 4.4 \| ≤ 0.0001 \| + \| + \|  \| \| Igf2^1^ \| 0.81 \| 0.02 \| 4.3 \| ≤ 0.0001 \|  \|  \| + \| \| Arc^1^ \| 0.83 \| 0.04 \| 5.6 \| ≤ 0.0001 \|  \|  \|  \| \| Meg3^1^ \| 0.81 \| 0.03 \| 4.6 \| ≤ 0.0001 \|  \|  \| + \| \| Rab3b^1^ \| 0.79 \| 0.03 \| 4.7 \| ≤ 0.0001 \|  \|  \|  \| \| Tcerg1l^1^ \| 0.70 \| 0.00 \| 6.1 \| ≤ 0.0001 \| + \| + \|  \| \| Pnmal2 \| 0.68 \| 0.01 \| 5.8 \| ≤ 0.0001 \|  \| + \|  \| \| Zcchc12 \| 0.67 \| 0.01 \| 5.6 \| ≤ 0.0001 \|  \|  \|  \| \| Chgb \| 0.71 \| 0.02 \| 6.2 \| ≤ 0.0001 \| + \| + \|  \| \| Ptn \| 0.84 \| 0.07 \| 3.3 \| ≤ 0.0001 \| + \| + \|  \| \| Rgs2 \| 0.76 \| 0.04 \| 4.4 \| ≤ 0.0001 \|  \|  \|  \| \| Cldn4 \| 0.89 \| 0.12 \| 4.5 \| ≤ 0.0001 \|  \|  \|  \| \| Meis2 \| 0.71 \| 0.03 \| 4.4 \| ≤ 0.0001 \| + \| + \|  \| \| Cdh13 \| 0.79 \| 0.07 \| 3.4 \| ≤ 0.0001 \|  \|  \|  \| \| Nrxn1 \| 0.76 \| 0.04 \| 3.1 \| ≤ 0.0001 \|  \|  \|  \| \| Scg2 \| 0.67 \| 0.03 \| 6.7 \| ≤ 0.0001 \| + \| + \|  \| \| Aplp1 \| 0.68 \| 0.03 \| 5.4 \| ≤ 0.0001 \|  \| + \|  \| \| Pcsk1n \| 0.62 \| 0.01 \| 4.7 \| ≤ 0.0001 \|  \|  \|  \| \| Ddc \| 0.63 \| 0.02 \| 5.2 \| ≤ 0.0001 \| + \| + \|  \| \| Ly6h \| 0.56 \| 0.00 \| 3.8 \| ≤ 0.0001 \| + \| + \|  \| \| St8sia3 \| 0.55 \| 0.00 \| 4.7 \| ≤ 0.0001 \|  \|  \|  \| \| Ptprn2 \| 0.65 \| 0.03 \| 4.7 \| ≤ 0.0001 \|  \|  \|  \| \| Cd9 \| 1.00 \| 0.87 \| 2.2 \| ≤ 0.0001 \| + \|  \|  \| \| Tspan13 \| 0.93 \| 0.24 \| 3.0 \| ≤ 0.0001 \|  \|  \|  \| \| Kcnip1 \| 0.56 \| 0.01 \| 5.3 \| ≤ 0.0001 \|  \|  \|  \| \| Egr1 \| 0.99 \| 0.56 \| 3.0 \| ≤ 0.0001 \|  \|  \|  \| \| Lrp11 \| 0.64 \| 0.04 \| 4.5 \| ≤ 0.0001 \| + \|  \|  \| \| Insm1 \| 0.53 \| 0.00 \| 3.9 \| ≤ 0.0001 \|  \| + \|  \| \| Peg3 \| 0.63 \| 0.04 \| 5.2 \| ≤ 0.0001 \|  \|  \|  \| \| Thbs1 \| 0.94 \| 0.32 \| 4.2 \| ≤ 0.0001 \|  \|  \|  \| \| Crmp1 \| 0.50 \| 0.00 \| 6.0 \| ≤ 0.0001 \|  \| + \|  \| \| Cldn9 \| 0.52 \| 0.00 \| 6.2 \| ≤ 0.0001 \|  \|  \|  \| \| Clstn2 \| 0.55 \| 0.01 \| 4.9 \| ≤ 0.0001 \|  \|  \|  \| \| Runx1t1 \| 0.53 \| 0.01 \| 4.7 \| ≤ 0.0001 \|  \| + \|  \| \| F5 \| 0.53 \| 0.01 \| 5.7 \| ≤ 0.0001 \|  \|  \|  \| \| *Ncam1 \| 0.53 \| 0.01 \| 4.9 \| ≤ 0.0001 \|  \|  \|  \| \| Prune2 \| 0.51 \| 0.01 \| 4.5 \| ≤ 0.0001 \|  \|  \|  \| \| Fhl2 \| 0.59 \| 0.03 \| 4.9 \| ≤ 0.0001 \|  \|  \|  \| \| Espn \| 0.74 \| 0.10 \| 3.2 \| ≤ 0.0001 \|  \|  \|  \| \| Scg3 \| 0.52 \| 0.01 \| 5.2 \| ≤ 0.0001 \|  \|  \|  \| \| Robo1 \| 0.66 \| 0.06 \| 3.5 \| ≤ 0.0001 \|  \|  \|  \| \| Zcchc18 \| 0.50 \| 0.01 \| 5.8 \| ≤ 0.0001 \|  \|  \|  \| \| Syp* \| 0.52 \| 0.01 \| 4.4 \| ≤ 0.0001 \|  \|  \|  \| \| Cpe \| 0.67 \| 0.07 \| 3.8 \| ≤ 0.0001 \|  \|  \|  \| \| Selm \| 0.59 \| 0.03 \| 3.2 \| ≤ 0.0001 \|  \|  \|  \| \| Krt18 \| 1.00 \| 0.76 \| 2.5 \| ≤ 0.0001 \|  \|  \|  \| \| Stxbp1 \| 0.66 \| 0.07 \| 4.1 \| ≤ 0.0001 \|  \|  \|  \| \| Olfm1 \| 0.50 \| 0.01 \| 4.8 \| ≤ 0.0001 \|  \|  \|  \| \| Bex1 \| 0.74 \| 0.12 \| 3.4 \| ≤ 0.0001 \|  \|  \|  \| \| Gnao1 \| 0.49 \| 0.01 \| 4.8 \| ≤ 0.0001 \|  \|  \|  \| \| Actg1 \| 1.00 \| 0.93 \| 2.1 \| ≤ 0.0001 \|  \|  \|  \| \| *Uchl1 \| 0.61 \| 0.04 \| 3.3 \| ≤ 0.0001 \| + \| + \|  \| \| Btg2 \| 0.99 \| 0.70 \| 2.5 \| ≤ 0.0001 \|  \|  \|  \| \| Nptx1 \| 0.49 \| 0.01 \| 6.1 \| ≤ 0.0001 \|  \|  \|  \| \| *Chga \| 0.63 \| 0.06 \| 5.8 \| ≤ 0.0001 \| + \| + \|  \| \| Syt7 \| 0.74 \| 0.12 \| 3.4 \| ≤ 0.0001 \| + \| + \|  \| \| Tnfrsf21 \| 0.72 \| 0.11 \| 3.1 \| ≤ 0.0001 \|  \|  \|  \| \| Dner \| 0.46 \| 0.00 \| 5.2 \| ≤ 0.0001 \|  \|  \|  \| \| Atf3 \| 0.94 \| 0.42 \| 3.4 \| ≤ 0.0001 \|  \|  \|  \| \| Egr3 \| 0.47 \| 0.01 \| 3.5 \| ≤ 0.0001 \|  \|  \|  \| \| Ptprz1 \| 0.77 \| 0.15 \| 3.3 \| ≤ 0.0001 \|  \|  \|  \| \| Itm2c \| 0.98 \| 0.57 \| 2.1 \| ≤ 0.0001 \|  \|  \|  \| \| Tmem171 \| 0.73 \| 0.13 \| 3.1 \| ≤ 0.0001 \|  \|  \|  \| \| Pcdh9 \| 0.46 \| 0.01 \| 5.1 \| ≤ 0.0001 \|  \|  \|  \| \| Wnt3 \| 0.43 \| 0.00 \| 5.1 \| ≤ 0.0001 \|  \|  \|  \| \| Insrr \| 0.44 \| 0.01 \| 5.1 \| ≤ 0.0001 \|  \| + \|  \| \| Disp2 \| 0.45 \| 0.01 \| 5.0 \| ≤ 0.0001 \|  \| + \|  \| \| Gpr126 \| 0.62 \| 0.06 \| 3.0 \| ≤ 0.0001 \|  \|  \|  \| \| Nr4a1 \| 0.89 \| 0.43 \| 3.7 \| ≤ 0.0001 \|  \|  \|  \| \| Tmod2 \| 0.46 \| 0.01 \| 4.6 \| ≤ 0.0001 \|  \|  \|  \| \| Smpd3 \| 0.73 \| 0.12 \| 2.9 \| ≤ 0.0001 \|  \|  \|  \| \| Nbl1 \| 0.84 \| 0.23 \| 2.4 \| ≤ 0.0001 \|  \|  \|  \| \| Ccnd2 \| 0.96 \| 0.45 \| 2.0 \| ≤ 0.0001 \|  \|  \|  \| \| Dmpk \| 0.56 \| 0.05 \| 3.9 \| ≤ 0.0001 \|  \|  \|  \| \| Stmn3 \| 0.42 \| 0.00 \| 5.1 \| ≤ 0.0001 \|  \|  \|  \| \| Sez6l2 \| 0.44 \| 0.01 \| 4.5 \| ≤ 0.0001 \|  \|  \|  \| \| Tspan7 \| 0.64 \| 0.09 \| 3.6 \| ≤ 0.0001 \|  \|  \|  \| \| 6330407J23Rik \| 0.39 \| 0.00 \| 4.7 \| ≤ 0.0001 \|  \|  \|  \| \| Cldn6 \| 0.45 \| 0.02 \| 4.0 \| ≤ 0.0001 \|  \|  \|  \| \| Cacna1h \| 0.41 \| 0.00 \| 3.8 \| ≤ 0.0001 \|  \|  \|  \| \| Mcoln3 \| 0.38 \| 0.00 \| 5.1 \| ≤ 0.0001 \|  \|  \|  \| \| Celf4 \| 0.50 \| 0.04 \| 4.1 \| ≤ 0.0001 \|  \|  \|  \| \| Krt8 \| 1.00 \| 0.86 \| 2.0 \| ≤ 0.0001 \|  \|  \|  \| \| Kcnk3 \| 0.47 \| 0.02 \| 3.6 \| ≤ 0.0001 \|  \|  \|  \| \| Cited4 \| 0.49 \| 0.03 \| 3.5 \| ≤ 0.0001 \|  \|  \|  \| |
| --- | --- | --- | --- | --- | --- | --- | --- | --- | --- | --- | --- | --- | --- | --- | --- | --- | --- | --- | --- | --- | --- | --- | --- | --- | --- | --- | --- | --- | --- | --- | --- | --- | --- | --- | --- | --- | --- | --- | --- | --- | --- | --- | --- | --- | --- | --- | --- | --- | --- | --- | --- | --- | --- | --- | --- | --- | --- | --- | --- | --- | --- | --- | --- | --- | --- | --- | --- | --- | --- | --- | --- | --- | --- | --- | --- | --- | --- | --- | --- | --- | --- | --- | --- | --- | --- | --- | --- | --- | --- | --- | --- | --- | --- | --- | --- | --- | --- | --- | --- | --- | --- | --- | --- | --- | --- | --- | --- | --- | --- | --- | --- | --- | --- | --- | --- | --- | --- | --- | --- | --- | --- | --- | --- | --- | --- | --- | --- | --- | --- | --- | --- | --- | --- | --- | --- | --- | --- | --- | --- | --- | --- | --- | --- | --- | --- | --- | --- | --- | --- | --- | --- | --- | --- | --- | --- | --- | --- | --- | --- | --- | --- | --- | --- | --- | --- | --- | --- | --- | --- | --- | --- | --- | --- | --- | --- | --- | --- | --- | --- | --- | --- | --- | --- | --- | --- | --- | --- | --- | --- | --- | --- | --- | --- | --- | --- | --- | --- | --- | --- | --- | --- | --- | --- | --- | --- | --- | --- | --- | --- | --- | --- | --- | --- | --- | --- | --- | --- | --- | --- | --- | --- | --- | --- | --- | --- | --- | --- | --- | --- | --- | --- | --- | --- | --- | --- | --- | --- | --- | --- | --- | --- | --- | --- | --- | --- | --- | --- | --- | --- | --- | --- | --- | --- | --- | --- | --- | --- | --- | --- | --- | --- | --- | --- | --- | --- | --- | --- | --- | --- | --- | --- | --- | --- | --- | --- | --- | --- | --- | --- | --- | --- | --- | --- | --- | --- | --- | --- | --- | --- | --- | --- | --- | --- | --- | --- | --- | --- | --- | --- | --- | --- | --- | --- | --- | --- | --- | --- | --- | --- | --- | --- | --- | --- | --- | --- | --- | --- | --- | --- | --- | --- | --- | --- | --- | --- | --- | --- | --- | --- | --- | --- | --- | --- | --- | --- | --- | --- | --- | --- | --- | --- | --- | --- | --- | --- | --- | --- | --- | --- | --- | --- | --- | --- | --- | --- | --- | --- | --- | --- | --- | --- | --- | --- | --- | --- | --- | --- | --- | --- | --- | --- | --- | --- | --- | --- | --- | --- | --- | --- | --- | --- | --- | --- | --- | --- | --- | --- | --- | --- | --- | --- | --- | --- | --- | --- | --- | --- | --- | --- | --- | --- | --- | --- | --- | --- | --- | --- | --- | --- | --- | --- | --- | --- | --- | --- | --- | --- | --- | --- | --- | --- | --- | --- | --- | --- | --- | --- | --- | --- | --- | --- | --- | --- | --- | --- | --- | --- | --- | --- | --- | --- | --- | --- | --- | --- | --- | --- | --- | --- | --- | --- | --- | --- | --- | --- | --- | --- | --- | --- | --- | --- | --- | --- | --- | --- | --- | --- | --- | --- | --- | --- | --- | --- | --- | --- | --- | --- | --- | --- | --- | --- | --- | --- | --- | --- | --- | --- | --- | --- | --- | --- | --- | --- | --- | --- | --- | --- | --- | --- | --- | --- | --- | --- | --- | --- | --- | --- | --- | --- | --- | --- | --- | --- | --- | --- | --- | --- | --- | --- | --- | --- | --- | --- | --- | --- | --- | --- | --- | --- | --- | --- | --- | --- | --- | --- | --- | --- | --- | --- | --- | --- | --- | --- | --- | --- | --- | --- | --- | --- | --- | --- | --- | --- | --- | --- | --- | --- | --- | --- | --- | --- | --- | --- | --- | --- | --- | --- | --- | --- | --- | --- | --- | --- | --- | --- | --- | --- | --- | --- | --- | --- | --- | --- | --- | --- | --- | --- | --- | --- | --- | --- | --- | --- | --- | --- | --- | --- | --- | --- | --- | --- | --- | --- | --- | --- | --- | --- | --- | --- | --- | --- | --- | --- | --- | --- | --- | --- | --- | --- | --- | --- | --- | --- | --- | --- | --- | --- | --- | --- | --- | --- | --- | --- | --- | --- | --- | --- | --- | --- | --- | --- | --- | --- | --- | --- | --- | --- | --- | --- | --- | --- | --- | --- | --- | --- | --- | --- | --- | --- | --- | --- | --- | --- | --- | --- | --- | --- | --- | --- | --- | --- | --- | --- | --- | --- | --- | --- | --- | --- | --- | --- | --- | --- | --- | --- | --- | --- | --- | --- | --- | --- | --- | --- | --- | --- | --- | --- | --- | --- | --- | --- | --- | --- | --- | --- | --- | --- | --- | --- | --- | --- | --- | --- | --- | --- | --- | --- | --- | --- | --- | --- | --- | --- | --- | --- | --- | --- | --- | --- | --- | --- | --- | --- | --- | --- | --- | --- | --- | --- | --- | --- | --- | --- | --- | --- | --- | --- | --- | --- | --- | --- | --- | --- | --- | --- | --- | --- | --- | --- | --- | --- | --- | --- | --- | --- | --- | --- | --- | --- | --- | --- | --- | --- | --- | --- | --- | --- | --- | --- | --- | --- | --- | --- | --- | --- | --- | --- | --- | --- | --- | --- | --- | --- | --- | --- | --- | --- | --- | --- | --- | --- | --- | --- | --- | --- | --- | --- | --- |

*Previously reported PNEC marker

^1^PNEC genes analyzed for expression in TNECs (Montoro et al, 2018)

^2^Non-neuroendocrine epithelial cells from mouse lung cell atlas (Travaglini et al, 2020)

^3^Natural log of the ratio of average expression (counts per million) in NE to non-PNEC epithelial cells

^4^Wilcoxon rank sum test

^5^Top reported mouse tracheal neuroendocrine cell (TNEC) marker genes in Montoro et al (2018)

^6^Top reported mouse TNEC marker genes in Plasschaert et al, 2018

^7^PNEC markers^1^ with little or no expression in TNECs (Montoro et al, 2018)
