# Supplementary material for: Neuroendocrinology of the lung revealed by single-cell RNA sequencing
Source: eLife. 2022 Dec 5;11:e78216. doi: 10.7554/eLife.78216 (PMC9721618; doi:10.7554/eLife.78216)
Supplement: Supplementary file 2. [file elife-78216-supp2.docx]

**Table S2: Extant PNEC markers**

| **Name** | **Gene** | **H** | **M** | **Other species detected** | **­Major extrapulmonary sites** |
| --- | --- | --- | --- | --- | --- |
| Chromogranin A | CHGA | Y | Y | Monkey, guinea pig^‡^ | Intestine, prostate NE cells |
| α-CGRP | CALCA | Y | Y | Guinea pig, rat, hamster, turtle, | Sensory neurons |
| Calcitonin | CALCA | Y | NA | Monkey, pig, dog, cat, guinea pig, rat, hamster, lizard | Thyroid (C-cells) |
| GRP/Bombesin | GRP | Y | N | Monkey, salamander | CNS, gastrointestinal tract |
| Substance P | Tac1 | Y | NA | Guinea pig, salamander | Neurons, CNS |
| NSE (Neuron specific enolase) | ENO2 | Y | NA | Monkey, cow, sheep, goat, rabbit, guinea pig, rat, hamster | CNS Neurons, peripheral NE cells |
| CCK | CCK | Y** | Y** | Rabbit**, frog**, guinea pig**, rat**, mouse**, hamster** | Intestine |
| PYY | PYY | NA | NA | Hamster | Endocrine pancreas, brain |
| Synapatophysin | SYP | Y | Y | Rabbit | Neurons |
| Protein gene product 9.5 (Pgp9.5) | UCHL1 | Y | Y | Monkey, sheep, cattle, goat, pig, dog, rabbit, guinea pig, rat, hamster, frog, toad, salamander^1^ | Neurons (pan-neuronal marker) |
| SV2 (Synaptic vesicle 2A) | SV2A | Y | Y^2^ | Rabbit^3^ | Neurons (central and peripheral) |
| Ascl1 (Achaete-scute homolog 1) | ASCL1 | Y | Y | NA | CNS and autonomic neurons |
| NCAM1 (neural cell adhesion molecule 1) | NCAM1 | Y | Y | Cat^4^ | Neurons (central and peripheral) |
| Insulinoma-associated protein-1 | INSM1^5^ | Y | Y | NA | Endocrine cells |
| Leu7/Hnk (CD 57) | B3GAT1 | Y | NA | Monkey | Embryonic multipotent neuroepithelial cells, NK cells |
| Somatostatin | SST | Y | NA | Monkey, cat, frog, toad^1^ | Neurons, |
| Enkephalin | PENK | Y | Y | Monkey, cat, guinea pig, rat, hamster, turtle, toad, | CNS neurons |
| Serotonin (5-HT) ^a^ | DDC, (TPH1,TPH2)^b^ | Y | Y | Monkey, rabbit, hamster | Neurons |
| Gad67 | GAD1^6^ | N | Y | NA | Brain and many other organs-major inhibitory neurotransmitter |

Nomenclature: a-CGRP (alpha-calcitonin gene related peptide), GRP (gastrin releasing peptide), Leu7/Hnk1 9 (Human natural killer-1): a carbohydrate epitope present on cell surface, PYY (peptide YY). Abbreviations: H: Human, M: Mouse, NA: Not assessed. Classic NE-specific markers were identified by targeted screening for molecules expressed in gut enteroendocrine cells or other neuroendocrine cells. ** equivocal or very scarce

^‡^ Reported previously, but without specific annotation whether chromogranin A or B

^a^ The synthetic genes for serotonin have not been previously reported to be expressed in PNECs, though serotonin has been detected. Thus, it is uncertain whether PNECs are capable of synthesizing serotonin or transporting it into the cell from extracellular sources.

^b^ Expression of these synthetic genes not previously assessed in pulmonary neuroendocrine cells

References

1. Polak, J. M. *et al.* Lung endocrine cell markers, peptides, and amines. *The Anatomical record* **236**, 169–171 (1993).

2. Cutz, E. Hyperplasia of pulmonary neuroendocrine cells in infancy and childhood. *Seminars in Diagnostic Pathology* **32**, 420–437 (2015).

3. Pan, J., Yeger, H. & Cutz, E. Innervation of pulmonary neuroendocrine cells and neuroepithelial bodies in developing rabbit lung. *Journal of Histochemistry & Cytochemistry* **52**, 379–389 (2004).

4. Seldeslagh, K. A. & Lauweryns, J. M. NCAM expression in the pulmonary neural and diffuse neuroendocrine cell system. *Microscopy Research and Technique* **37**, 69–76 (1997).

5. Jia, S., Wildner, H. & Birchmeier, C. Insm1 controls the differentiation of pulmonary neuroendocrine cells by repressing Hes1. *Developmental Biology* **408**, 90–98 (2015).

6. Schnorbusch, K. *et al.* GABAergic signaling in the pulmonary neuroepithelial body microenvironment: functional imaging in GAD67-GFP mice. *Histochemistry and Cell Biology* **140**, 549–566 (2013).
