# Supplementary material for: Neuroendocrinology of the lung revealed by single-cell RNA sequencing
Source: eLife. 2022 Dec 5;11:e78216. doi: 10.7554/eLife.78216 (PMC9721618; doi:10.7554/eLife.78216)
Supplement: Supplementary file 3. [file elife-78216-supp3.docx]

**Table S3. Expression of neurotransmitter biosynthetic, vesicular loading and reuptake genes in mouse PNECs^1^**

|  |  | **Synthesis (S)** | | **Vesicular transport (V_T_)** | | **Reuptake (R)** | |
| --- | --- | --- | --- | --- | --- | --- | --- |
| **Neurotransmitter** | **Sum** | **Genes (%NE^+^)** | **S** | **Genes (%NE^+^)** | **V_T_** | **Genes (%NE^+^)** | **R** |
| Serotonin | + | Tph1 (0%),  Tph2 (0%),  Ddc (63%) | - | Slc18a1/VMAT1 (46%), Slc18a2 /VMAT2 (1%) | + | **Slc6a4** (24%) | + |
| GABA | + | **Gad1** (28%),  Gad2 (0%) | + | Slc32a1/VIAAT (0%) | - | Slc6a1 (0%), Slc6a11 (0%), Slc6a13 (0%),  Slc6a12 (0%) | - |
| Glutamate | + | Gls (31%),  Gls2 (0%) | + | Slc17a7 (0%),  **Slc17a6** (1%),  Slc17a8 (90%) | + | Slc1a1 (0%),  Slc1a2 (0%),  Slc1a3 (0%),  Slc1a6 (0%),  Slc1a7 (0%) | - |
| Dopamine | + | **Th** (1%),  Ddc (63%) | + | Slc18a1/VMAT1 (46%),  Slc18a2 /VMAT2 (1%) | + | Slc6a3 (0%) | - |
| Norepinephrine | - | Th (1%),  Ddc (63%),  Dbh (0%) | - | Slc18a1/VMAT1 (46%),  Slc18a2 /VMAT2 (1%) | + | Slc6a2 (1%) | + |
| Epinephrine | - | Th (1%),  Ddc (63%),  Dbh (0%),  Pnmt (0%) | - | Slc18a1/VMAT1(46%),  Slc18a2 /VMAT2 (1%) | + | Slc6a2 (1%) | + |
| Acetylcholine | - | Chat (0%) | - | **Slc18a3**/VAChT (1%) | + | Slc5a7 (0%), Slc44a4 (0%) | - |
| Glycine | - | NA | NA | Slc32a1/VIAAT | - | Slc6a9 (16%, NS), Slc6a5 (0%) | (+) |
| Histamine | + | **Hdc** (1%) | + | Slc18a1/VMAT1 (46%),  Slc18a2 /VMAT2 (1%) | + | NA | NA |

Sum (Summary), overall inferred activity of neurotransmitter pathway. +, expressed neurotransmitter or gene (percent of PNECs expressing); (+), some but not all genes in biosynthetic pathway detected; -, gene(s) not detected. Bold font, genes unique to neurotransmitter pathway. Red, genes not previously known to be expressed. Tph1, Tryptophan hydroxylase 1; Tph2, Tryptophan hydroxylase 2; Ddc, Dopamine decarboxylase; Gad1, Glutamate decarboxylase 1, Gad2, Glutamate decarboxylase 2; VMAT1, vesicular monoamine transporter 1; VMAT2, vesicular monoamine transporter 2. VIAAT, vesicular inhibitory amino acid transporter. NA, not applicable; NS, not selectively expressed in PNECs.

Neurotransmitter pathway genes curated from Shammas NK, Hung Y-T, Wang Z-W, editors (2008)

Neurotransmitter reuptake and synaptic vesicle refilling. In Molecular Mechanisms of Neurotransmitter

Release, Contemporary Neuroscience Series (Human Press), p. 264-293.
