# Supplementary material for: Neuroendocrinology of the lung revealed by single-cell RNA sequencing
Source: eLife. 2022 Dec 5;11:e78216. doi: 10.7554/eLife.78216 (PMC9721618; doi:10.7554/eLife.78216)
Supplement: Supplementary file 4. [file elife-78216-supp4.docx]

**Table S4. Summary of mouse and human peptidergic gene expression in PNECs**

| **Name** | **Mouse** | **Human** | **Class** | **M1 (%)** | **M2 (%)** | **Mc (%)** | **H (%)** | ***p-value** |
| --- | --- | --- | --- | --- | --- | --- | --- | --- |
| **Pituitary adenylcyclase-activated peptide** | **Adcyap1** | **ADCYAP1** | **NP** | **2** | **1** | **2** | **43** | 0.7 |
| Adiponectin | Adipoq | ADIPOQ | Hormone | 0 | 0 | 0 | 0 |  |
| **Adrenomedullin­** | **Adm** | **ADM** | **Hormone** | **1** | **5** | **2** | **6** | 0.05 |
|  | Adm2 | ADM2 | Hormone | 0 | 0 | 0 | 0 |  |
| **Agouti related neuropeptide** | **Agrp** | **AGRP** | **NP** | **1** | **0** | **1** | **4** | 0.5 |
| **Angiotensinogen** | **Agt** | **AGT** | **Hormone** | **16** | **11** | **14** | **4** | 0.4 |
| Apelin | Apln | APLN | NP | 0 | 0 | 0 | 0 |  |
| Anti-mullerian hormone | Amh | AMH | Hormone | 0 | 0 | 0 | 17 |  |
| Vasopressin | Avp | AVP | Hormone | 0 | 0 | 0 | 0 |  |
| Bone gamma-carboxyglutamic acid-containing protein | Bglap | BGLAP | Hormone | 0 | 0 | 0 | 0 |  |
| **Calcitonin/CGRP** | **Calca** | **CALCA** | **Hormone/NP** | **95** | **97** | **93** | **91** | 0.8 |
| **Calcitonin related peptide beta** | **Calcb** | **CALCB** | **NP** | **13** | **28** | **18** | **11** | 0.004 |
| **Cocaine and amphetamine-related transcript** | **Cartpt** | **CARTPT** | **NP** | **34** | **24** | **29** | **4** | 0.1 |
| Cholecystokinin | Cck | CCK | Hormone | 0 | 0 | 0 | 4 |  |
| **Chromogranin A** | **Chga** | **CHGA** | **NP** | **60** | **54** | **56** | **91** | 0.4 |
| **Chromogranin B** | **Chgb** | **CHGB** | **NP** | **70** | **77** | **70** | **83** | 0.2 |
| Glycoprotein hormones, subunit A | Cga | CGA | NP | 0 | 0 | 0 | 19 |  |
| Cortistatin | Cort | CORT | NP | 0 | 0 | 0 | 0 |  |
| Corticotropin releasing hormone | Crh | CRH | Hormone | 1 | 1 | 1 | 0 | 1 |
| **Diazepam binding inhibitor** | **Dbi** | **DBI** | **NP** | **27** | **33** | **28** | **65** | 0.4 |
| **Endothelin I** | **Edn1** | **EDN1** | **Hormone** | **0** | **7** | **3** | **4** | 0.001 |
| Endothelin II | Edn2 | EDN2 | Hormone | 0 | 0 | 0 | 0 |  |
| Endothelin III | Edn3 | END3 | Hormone | 1 | 1 | 1 | 0 | 1 |
| Erythropoietin | Epo | EPO | Hormone | 0 | 0 | 0 | 2 |  |
| Follicle stimulating hormone, subunit beta | Fshb | FSHB | Hormone | 0 | 0 | 0 | 0 |  |
| Galanin | Gal | GAL | NP | 1 | 7 | 3 | 0 | 0.02 |
| Galanin like-peptide | Galp | GALP | NP | 0 | 0 | 0 | 0 |  |
| Gastrin | Gast | GAST | Hormone | 0 | 0 | 0 | 0 |  |
| Glucagon | Gcg | GCG | Hormone | 0 | 0 | 0 | 0 |  |
| Growth hormone | Gh | GH | Hormone | 0 | 0 | 0 | 0 |  |
|  | na | GH2 | Hormone | na | na | na | 0 |  |
| Growth hormone releasing hormone | Ghrh | GHRH | Hormone | 0 | 0 | 0 | 0 |  |
| **Ghrelin** | **Ghrl** | **GHRL** | **Hormone** | **0** | **2** | **1** | **6** | 0.1 |
| Gastric inhibitory peptide | Gip | GIP | Hormone | 0 | 0 | 0 | 0 |  |
| **Gonadotropin Releasing Hormone 1** | **Gnrh1** | **GNRH1** | **Hormone** | **1** | **3** | **2** | **2** | 0.3 |
| Gonadotropin Releasing Hormone 2 | na | GNRH2 | Hormone | na | na | 0 | 4 |  |
| Gastrin-releasing peptide | Grp | GRP | Hormone | 0 | 0 | 0 | 98 |  |
| Hepcidin | Hamp | HAMP | Hormone | 0 | 0 | 0 | 0 |  |
| **Hypocretin** | **Hcrt** | **HCRT** | **NP** | **1** | **1** | **1** | **4** | 1 |
| Islet amyloid polypeptide (Amylin) | Iapp | IAPP | Hormone | 7 | 9 | 7 | 0 | 0.6 |
| **Inhibin A** | **Inha** | **INHA** | **Hormone** | **18** | **17** | **17** | **6** | 1 |
| **Inhibin, subunit beta A** | **Inhba** | **INHBA** | **Hormone** | **0** | **9** | **3** | **30** | 0.0002 |
| **Inhibin, subunit beta B** | **Inhbb** | **INHBB** | **Hormone** | **5** | **0** | **3** | **17** | 0.0 |
| Insulin like growth factor 1 | Igf1 | IGF1 | Hormone | 0 | 1 | 0 | 0 | 0.3 |
| Insulin like growth factor 2 | Igf2 | IGF2 | Hormone | 81 | 64 | 72 | 0 | 0.003 |
| Insulin | Ins1 | INS | Hormone | 0 | 0 | 0 | 0 |  |
| Luteinizing hormone (subunit beta) | Lhb | LHB | Hormone | 0 | 1 | 0 | 0 | 0.3 |
| Kininogen | Kng1 | KNG1 | NP | 0 | 0 | 0 | 4 |  |
| Leptin | Lep | LEP | Hormone | 0 | 0 | 0 | 0 |  |
| Motilin | na | MLN | Hormone | na | na | na | 0 |  |
| **Neuromedin B** | **Nmb** | **NMB** | **NP** | **1** | **1** | **1** | **11** | 1 |
| Neuromedin S | Nms | NMS | NP | 0 | 0 | 0 | 0 |  |
| Neuromedin U | Nmu | NMU | NP | 0 | 0 | 0 | 0 |  |
| Neuropeptide B | Npb | NPB | NP | 0 | 1 | 0 | 0 | 0.3 |
| Neuropeptide amide FF | Npff | NPFF | NP | 1 | 1 | 1 | 0 | 1.0 |
| **Atrial natriuretic peptide (ANP)** | **Nppa** | **NPPA** | **Hormone** | **1** | **1** | **1** | **14** | 1.0 |
| Natriuretic peptide, type B | Nppb | NPPB | Hormone | 0 | 1 | 0 | 0 | 0.3 |
| Natriuretic peptide, type C | Nppc | NPPC | Hormone | 0 | 1 | 0 | 0 | 0.3 |
| Neuropeptide S | Nps | NPS | NP | 0 | 0 | 0 | 0 |  |
| **Neuropeptide W** | **Npw** | **NPW** | **NP** | **1** | **1** | **1** | **13** | 1 |
| Neuropeptide Y | Npy | NPY | NP | 0 | 0 | 0 | 4 |  |
| Neurotensin | Nts | NTS | NP | 0 | 0 | 0 | 0 |  |
| Osteocrine | Ostn | OSTN | NP | 0 | 0 | 0 | 0 |  |
| Oxytocin | Oxt | OXT | Hormone/NP | 0 | 5 | 2 | 0 | 0.004 |
| **Proprotein convertase subtilisin/kexin type 1 inhibitor** | **Pcsk1n** | **PCSK1N** | **NP** | **62** | **14** | **43** | **100** | < 0.001 |
| Prodynorphin | Pdyn | PDYN | NP | 0 | 0 | 0 | 0 |  |
| Proenkephalin | Penk | PENK | NP | 0 | 0 | 0 | 0 |  |
| Pro-melanin concentrating hormone | Pmch | PMCH | NP | 0 | 0 | 0 | 0 |  |
| Prepronociceptin | Pnoc | PNOC | NP | 0 | 9 | 3 | 0 | 0.002 |
| **Pro-opiomelanocortin-alpha** | **Pomc** | **POMC** | **NP** | **2** | **7** | **4** | **57** | 0.1 |
| Pancreatic polypeptide | Ppy | PPY | NP | 0 | 0 | 0 | 0 |  |
| Prolactin | Prl | PRL | Hormone | 0 | 0 | 0 | 0 |  |
| Prolactin releasing hormone | Prlh | PRLH | Hormone | 0 | 0 | 0 | 2 |  |
| Prokinectin 1 | Prok1 | PROK1 | NP | 0 | 0 | 0 | 0 |  |
| Prokinectin 2 | Prok2 | PROK2 | NP | 0 | 4 | 1 | 0 | 0.01 |
| Parathryroid hormone | Pth | PTH | Hormone | 0 | 0 | 0 | 0 |  |
| Parathyroid hormone like hormone | Pthlh | PTHLH | Hormone | 0 | 0 | 0 | 0 |  |
| Peptide YY | Pyy | PYY | NP | 0 | 0 | 0 | 0 |  |
| Neuropeptide VF precursor | Npvf | NPVF | NP | 0 | 0 | 0 | 0 |  |
| Relaxin | Rln1 | RLN1 | Hormone | 0 | 0 | 0 | 0 |  |
| Renin | Ren1 | REN | Hormone | 0 | 0 | 0 | 2 |  |
| Resistin | Retn | RETN | Hormone | 0 | 0 | 0 | 0 |  |
| **Secretogranin 2** | **Scg2** | **SCG2** | **NP** | **66** | **82** | **70** | **96** | 0.007 |
| **Secretogranin 3** | **Scg3** | **SCG3** | **NP** | **51** | **70** | **56** | **94** | 0.004 |
| **Secretogranin 5** | **Scg5** | **SCG5** | **NP** | **95** | **95** | **92** | **98** | 1 |
| Secretin | Sct | SCT | Hormone | 0 | 0 | 0 | 0 |  |
| Somatostatin | Sst | SST | Hormone | 0 | 0 | 0 | 6 |  |
| Tachykinin 1 | Tac1 | TAC1 | NP | 0 | 0 | 0 | 0 |  |
| Thrombopoietin | Thpo | THPO | Hormone | 1 | 4 | 2 | 0 | 0.2 |
| Thyrotropin releasing hormone | Trh | TRH | Hormone | 0 | 0 | 0 | 43 |  |
| Urocortin | Ucn | UCN | Hormone/NP | 0 | 0 | 0 | 2 |  |
| Urocortin 2 | Ucn2 | UCN2 | Hormone | 0 | 36 | 13 | 0 | 1 |
| Urocortin 3 | Ucn3 | UCN3 | Hormone | 0 | 0 | 0 | 0 |  |
| Urotensin 2 | Uts2 | UTS2 | NP/Hormone | 1 | 1 | 1 | 0 | 1 |
| Urotensin 2b | na | UTS2B | NP/Hormone | 0 | 0 | 0 | 2 |  |
| **Vgf nerve growth factor** | **Vgf** | **VGF** | **NP** | **19** | **33** | **23** | **31** |  |
| Vasoactive intestinal peptide | Vip | VIP | Hormone | 0 | 0 | 0 | 0 |  |
| Total number of genes | 93 | 97 |  | 31 | 41 | 43 | 40 |  |

| **Genes detected in mouse and human;** Genes detected only in mouse; Genes detected only in human; Genes not detected in mouse or human  M1, Mus musculus data set 1 (n = 176 PNECs); M2, Mus musculus data set 2 (n = 92 PNECs); Mc, Mus musculus data sets 1 and 2 combined (n = 268 PNECs)  H, Homo sapiens (n = 55 PNECs)  NP, neuropeptide; na, not applicable  *Comparison between M1 and M2, Fisher's exact test (two-tailed) |
| --- |
