# Supplementary material for: Neuroendocrinology of the lung revealed by single-cell RNA sequencing
Source: eLife. 2022 Dec 5;11:e78216. doi: 10.7554/eLife.78216 (PMC9721618; doi:10.7554/eLife.78216)
Supplement: Supplementary file 5. [file elife-78216-supp5.docx]

**Table S5. PNEC peptidergic genes, receptors, and classic functions**

| **Gene** | **Gene name** | **Peptides produced** | **Receptor genes** | **Major sites of NP expression (function)** |
| --- | --- | --- | --- | --- |
| ADCYAP1 | Pituitary adenylcyclase-activated peptide | PACAP-38, PACAP-27, PRP-48  (alternative splicing 🡪 many transcript variants) | ADCYAP1R1 | CNS neurons, enteroendocrine cells (diverse physiologic functions) |
| ADM | Adrenomedullin | Adrenomedullin, Am, Pamp | CALCRL, RAMP3 | Vascular endothelial cells (vasodilation, hypotensive peptide). Originally isolated from pheochromocytoma (tumor of adrenal medulla). |
| AGRP | Agouti-related peptide | Agrp | MC3R, MC4R | Neurons, adrenal medulla, hypothalamus (adipose tissue regulation) |
| AGT | Angiotensinogen | Angiotensin I, Angiotensin II (9 aa), Angiotensin (1-7) | (*Agtr1a*)  (*Agtr1b*)  AGTR1 | Kidneys (proximal tubule), regulation of systemic blood pressure |
| CALCA* | Calcitonin related polypeptide pre-protein alpha | Calcitonin gene related peptide I (α-CGRP) (37 aa), Calcitonin, Katacalcin, | CALCRL, RAMP1, CALCRL, RAMP3 (lower affinity)  CALR | Sensory neurons (vasodilation in peripheral vasculature)  Thyroid gland C-cells (calcium homeostasis) |
| CALCB | Calcitonin related polypeptide beta | CGRP II (b-CGRP) | CALCRL, RAMP1, RAMP2, RAMP3 | Unknown |
| CARTPT | Cocaine and amphetamine-related transcript | CART- (1-39), CART- (42-89) | GPR68 | Central and peripheral neuron terminals (regulates cell homeostasis, multiple pathways, regulates insulin) |
| CCK | Cholecystokinin | Cholecystokinin | CCKAR, CCKBR | Gastrointestinal tract (regulates gastric motility) |
| CHGA* | Chromogranin A | Chromogranin A, beta-granin, vasostatin 1, vasostatin 2, pancreastatin, EA | nd | CNS and peripheral neurons, endocrine and neuroendocrine cells (formation of secretory granules and packaging hormones in NE cells); vasostatins (cardiovascular homeostasis, inhibits vascular tone) |
| CHGB** | Chromogranin B | Chromogranin B (secretogranin I), CCB peptide, GAWK peptide | nd | CNS, Endocrine tissues (secretory granule packaging) |
| CRH | Corticotropin releasing hormone | Crh | CRHR1, CRHR2 | CNS neurons, pituitary neuroendocrine cells (stimulates synthesis, release of ACTH) |
| DBI | Diazepam binding inhibitor | Diazepam binding inhibitory peptide | nd | Intestine (endogenous ligand of type A GABA receptors. regulates secretion of cholecystokinin (CCK)) |
| EDN1 | Endothelin I | Endothelin-1 | EDNRA | Endothelial cells (vasoconstriction) |
| EDN3 | Endothelin III | Endothelin-3 | EDNRB | possible function in development of neural crest derivatives. Mutations in receptor gene EDNRB associated with Hirshprung disease and Waardenburg syndrome in humans. |
| GAL | Galanin | Galanin, GAMP, Galp, alarin | GALR1 | Central and peripheral neurons, gut (implicated in regulation of multiple physiologic functions: nociception, cognition, feeding, blood pressure) |
| GHRL | Ghrelin and Obestatin pre-propeptide | Grehlin and obestatin | GHSR | Stomach, duodenum: Ghrelin (secreted by stomach) is an appetite stimulant. Obestatin regulates multiple metabolic activities |
| GNRH1 | Gonadotropin Releasing Hormone 1 | Gonadotropin Releasing Hormone 1 | GNRHR | Hypothalamus, stimulates synthesis and secretion of gonadotropins (FSH and LH) |
| GNRH2 | Gonadotropin-releasing hormone 2 | Gonadotropin-releasing hormone 2 | GNRHR |  |
| GRP | Gastrin-releasing peptide | Gastrin releasing peptide -alternate splicing | GRPR | Stomach (stimulates gastrin release from G cells, also a potent mitogen) |
| HCRT | Hypocretin | Hypocretin1, hypocretin-2, (orexin-1, orexin-2) | HCRT1, HCRT2 | CNS neurons (regulates arousal, wakefulness, appetite) |
| IAPP | Islet amyloid polypeptide | Amylin, Amyloid polypeptide | CALCR, RAMP1, RAMP2, RAMP3 | Pancreas (β-cells), co-secreted with insulin and functions in glycemic control |
| IGF1 | Insulin-like growth factor 1 | Somatomedin-C | IGF1R | Broad expression (cell growth, insulin-like effects |
| IGF2 | Insulin-like growth factor 2 | Igf-2 | IGF1R, IGF2R | Growth promoting hormone |
| INHA | Inhibin alpha subunit | Subunit of inhibin | ACVR2A, ACVR2B, ACVR1, ACVR1B, ACVR1C | Activin receptors |
| INHBA | Inhibin, beta a | Subunit of activin and inhibin | TGFBR3 | Gonadal tissues (inhibits FSH production (targets pituitary), inhibin secreted by Sertoli cells (testes), granulosa cells (ovary), trophoblasts (fetus) |
| INHBB | Inhibin, beta b |  |  |  |
| KNG1 | Kininogen | Kininogen-1 (alpha-2 thiol proteinase inhibitor), alternate splicing 🡪 HMWK (644 aa) and LMWK (427 aa), bradykinin (9 aa) | BDKRB1 | HMWK: Blood coagulation  bradykinin: antimicrobial peptide |
| LHB | Leutinizing hormone | Leutinizing hormone | LHCGR | Pituitary (increase sex hormone production targets testes/ovaries) |
| NMB | Neuromedin B | Nmb | NMBR | Diverse functions including regulating exocrine, endocrine secretion, cell growth, blood pressure |
| NPB | Neuropeptide B | Neuropeptide B23, neuropeptide B29 | NPBWR1 (GPR7), NPBWR2 (GRP8) | Expressed in kidney, fat. Regulate feeding, pain perception |
| NPFF | Neuropeptide FF | Neuropeptide FF1, neuropeptide AF, neuropeptide SF, | NPFFR11, NPFFR2 | CNS, nociception |
| NPPA | Natriuretic Peptide A | Atrial natriuretic factor (natriuretic peptide A, ANF, ANP, natriodilantine), | NPR1, NPR3 | Heart (atria), potent inhibitor of renal tubular  NaCl reabsorption |
| NPPB | Brain natriuretic peptide (B-type natriuretic peptide) | NT-proBNP | NPR1 | Hormone secreted by ventricular cardiomyocytes in response to stretching, decreases systemic vascular resistance |
| NPPC | Natriuretic peptide (C-type natriuretic peptide) | C-type natriuretic peptide | NPR2 | Potent natriuretic, diuretic, and vasodilator |
| NPW | Neuropeptide W | Neuropeptide W pro-peptide, NPW-27 | NPBWR1 (GPR7), NPBWR2 (GRP8) | Hypothalamus, regulates catabolic functions, vascular tone |
| OXT | Oxytocin | Oxytocin | OXTR | Receptors expressed by myoepithelial cells of mammary gland, myometrium and endometrium in pregnancy to induce lactation and uterine contractions at end of pregnancy |
| NPY | Neuropeptide Y | Pro-neuropeptide Y, Neuropeptide Y | NPY1R, NPY2R, NPY4R, NPY4R2, NPY5R | Distributed throughout CNS and PNS with expression mainly in post-ganglionic sympathetic neurons. Most abundant NP in the heart. |
| PCSK1N | Proprotein convertase subtilisin/kexin type 1 inhibitor | [ProSAAS] BigLEN, PEN, Little SAAS, LittleLEN | GPR171 –receptor for BigLEN | CNS (secretory pathway protein and neuropeptide precursor, inhibits activity of Pcsk1) |
| PNOC | Prepronociceptin | Nociceptin (orphanin FQ2), nocistatin | OPRL1 | CNS, intestine, vas deferens, spleen and immune system  (nociception, motor and balance, stress response, sexual behavior, and other diverse physiologic functions) |
| **POMC**** | Pro-opiomelanocortin | α-MSH (α-melanocyte-stimulating hormone), adrenocorticotropic hormone (ACTH), corticotropin-like intermediate peptide (CLIP), β-lipotropin (β-LPH), γ-Lipotropin (γ-LPH), β-endorphin, α-endorphin, γ-endorphin, | MC2R (ACTH)  MC1R (MSH), MC3R, MC4R, MC5R  Mu-opioid receptors (OPRM1)  Met-enkephalin receptors (OPRK1) | CNS neurons (pituitary gland), ACTH key regulator of cortisol release (essential component of the hypothalamic-pituitary-adrenal) axis. MSH produced by keratinocytes in skin for pigmentation in response to UV light. |
| PRLH | Prolactin releasing hormone | Prolactin releasing hormone | PRLHR | Hypothalamus: stimulates prolactin release |
| PROK2 | Prokinectin 2 | Prokinectin 2 | PROKR2 | Contracts gastrointestinal smooth muscle, but also wide variety of physiologic functions. |
| SCG2** | Secretogranin II | Chromogranin C (secretogranin II), secretoneurin, EM66 | nd | Neurondocrine cells and CNS –pituitary, hypothalamus (multiple reported functions: eosinophil chemoattractant, released from variety of neuroendocrine tissues in response to depolarization by capsaicin) |
| SCG3** | Secretogranin III | Secretogranin III | nd | Neuroendocrine, endocrine cells (secretory vesicle packaging), localized to secretory vesicles and interacts with Chga. |
| SCG5 | Secretogranin 5 (Neuroendocrine protein 7B2 precursor) | N-terminal peptide (27-126), C-terminal peptide (200-212) | nd | CNS, neuroendocrine tissues |
| SST | Somatostatin | Somatostatin (Growth hormone inhibiting hormone) –peptide hormone | SSTR1, SSTR2,SSTR3,SSTR4, SSTR5 | Hypothalamus: regulates GH secretion |
| THPO | Thrombopoietin | Thrombopoietin | MPL | Thyroid hormone synthesis, thyroid gland |
| TRH | Thyrotropin-releasing hormone | TRH | TRHR | Hypothalamus: regulates TSH and prolactin release |
| UCN | Urocortin | Urocortin | CRHR1, CRHR2 | Supraoptic nucleus, hippocampus, Stress response |
| UCN2 | Urocortin 2 | Urocortin 2 | CRHR1, CRHR2 | CRF family of peptides, autonomic and appetite control. |
| UTS2 | Urotensin 2 | Urotensin-2 | UTS2R | CNS neurons and diverse tissues: kidney, spleen, small intestine, thymus, prostate, pituitary, and adrenal gland and circulates in human plasma; most potent vasoconstrictor |
| UTS2B | Urotensin 2b | Urotensin-2B (paralog of UTS2) | UTS2R | CNS and peripheral tissues. Cardiovascular system, and renal system in humans, with reported vasoactive function |
| VGF | Vgf nerve growth factor inducible protein | Vgf, TLPQ-62, AQEE-30, LQEQ-19 | C1QBP | Widely expressed in nervous system, including peripheral endocrine and neuroendocrine tissues. Diverse physiologic roles reported (in energy balance, pain, fluid balance, reproduction). |

*Genes previously identified in PNECs, **Genes expressed in human NE tumors but not previously reported in normal lung. Only human gene symbols (capitalized) are shown (see Table S4 for corresponding

mouse genes)*.* GRP and NMB, mammalian homologs of bombesin, previously reported in human PNECs. Blue, classic hormones. nd, not determined.
