# Supplementary material for: Neuroendocrinology of the lung revealed by single-cell RNA sequencing
Source: eLife. 2022 Dec 5;11:e78216. doi: 10.7554/eLife.78216 (PMC9721618; doi:10.7554/eLife.78216)
Supplement: Supplementary file 8. [file elife-78216-supp8.docx]

**Table S8. Expression of neurotransmitter biosynthetic, vesicular loading and reuptake genes in human PNECs^1^**

|  |  | **Synthesis** | | **Vesicular transport (V_T_)** | | **Reuptake** | |
| --- | --- | --- | --- | --- | --- | --- | --- |
| **Neurotransmitter** | **Sum** | **Genes (%NE^+^)** | **S** | **Genes (%NE^+^)** | **V_T_** | **Genes (%NE^+^)** | **R** |
| Serotonin | + | **TPH1**(76%), TPH2 (0%),  DDC (96%) | + | SLC18A1/VMAT1 (24%), SLC18A2/VMAT2 (2%) | (+) | SLC6A4 (0%) | - |
| GABA | + | **GAD1** (13%), GAD2 (0%) | + | SLC32A1/VIAAT (2%) | + | **SLC6A1** (2%) SLC6A11, SLC6A12,  **SLC6A13** (2%) | + |
| Glutamate | + | GLS (17%),  GLS­2 (10%) | + | SLC17A7, **SLC17A6** (14%), SLC17A8 (0%) | + | **SLC1A1**, **SLC1A2** (4%), SLC1A3, SLC1A6, SLC1A7 | (+) |
| Dopamine | - | TH, DDC (96%) | - | SLC18A1/VMAT1 (24%), SLC18A2/VMAT2 (2%) | (+) | SLC6A3 | - |
| Norepinephrine | (+) | TH, DDC (96%),  DBH (4%) | (+) | SLC18A1/VMAT1 (24%), SLC18A2/VMAT2 (2%) | (+) | SLC6A2 | - |
| Epinephrine | (+) | TH, DDC (96%),  DBH (4%),  PNMT (6%) | (+) | SLC18A1/VMAT1 (24%), SLC18A2/VMAT2 (2%) | (+) | SLC6A2 | - |
| Acetylcholine | - | CHAT | - | SLC18A3/VAChT | - | SLC5A7, SLC44A4 | - |
| Glycine | + | NA | (+) | SLC32A1/VIAAT (2%) | + | SLC6A9,  **SLC6A5 (**72%) | + |
| Histamine | - | HDC | - | SLC18A1/VMAT1 (24%), SLC18A2/VMAT2 (2%) | (+) | NA | NA |

Sum (Summary), overall inferred activity of neurotransmitter pathway. +, expressed gene (percent of PNECs expressing); (+), some but not all genes in biosynthetic pathway detected; -, gene not detected. Bold font, genes unique to neurotransmitter pathway. Red, genes not previously known to be expressed. TPH1, Tryptophan hydroxylase 1; TPH2, Tryptophan hydroxylase 2; DDC, Dopamine decarboxylase; GAD1, Glutamate decarboxylase 1; GAD2, Glutamate decarboxylase 2; GLS2, Glutaminase2; VMAT1, vesicular monoamine transporter 1; VMAT2, vesicular monoamine transporter 2. VIAAT, vesicular inhibitory amino acid transporter. NA, not applicable.

Neurotransmitter pathway genes curated from Shammas NK, Hung Y-T, Wang Z-W, editors (2008)

Neurotransmitter reuptake and synaptic vesicle refilling. In Molecular Mechanisms of Neurotransmitter

Release, Contemporary Neuroscience Series (Human Press), p. 264-293.
